# The Development and Evaluation of “Life Age”—a Primary Prevention and Population-Focused Risk Communication Tool: Feasibility Study With a Single-Arm Repeated Measures Design

**DOI:** 10.2196/37385

**Published:** 2022-10-24

**Authors:** Adeogo Akinwale Olusan, Suzanne Barr, Mark Cobain, Holly Whelan

**Affiliations:** 1 Department of Cardiology Belfast Health and Social Care (HSC) Trust Belfast United Kingdom; 2 Faculty of Medicine National Heart and Lung Institute Imperial College London London United Kingdom; 3 Younger Lives Limited London United Kingdom

**Keywords:** Life Age, Heart Age, cardiovascular risk, lifestyle change, psychosocial well-being, population focused risk communication tool, health promotion, risk perception, premature mortality, risk communication

## Abstract

**Background:**

Communicating cardiovascular risk to the general population requires forms of communication that can enhance risk perception and stimulate lifestyle changes associated with reduced cardiovascular risk.

**Objective:**

The aim of this study was to evaluate the motivational potential of a novel lifestyle risk assessment (“Life Age”) based on factors predictive of both premature mortality and psychosocial well-being.

**Methods:**

A feasibility study with a single-arm repeated measures design was conducted to evaluate the potential efficacy of Life Age on motivating lifestyle changes. Participants were recruited via social media, completed a web-based version of the Life Age questionnaire at baseline and at follow-up (8 weeks), and received 23 e-newsletters based on their Life Age results along with a mobile tracker. Participants’ estimated Life Age scores were analyzed for evidence of lifestyle changes made. Quantitative feedback of participants was also assessed.

**Results:**

In total, 18 of 27 participants completed the two Life Age tests. The median baseline Life Age was 1 year older than chronological age, which was reduced to –1.9 years at follow-up, representing an improvement of 2.9 years (*P*=.02). There were also accompanying improvements in Mediterranean diet score (*P*=.001), life satisfaction (*P*=.003), and sleep (*P*=.05). Quantitative feedback assessment indicated that the Life Age tool was easy to understand, helpful, and motivating.

**Conclusions:**

This study demonstrated the potential benefit of a novel Life Age tool in generating a broad set of lifestyle changes known to be associated with clinical risk factors, similar to “Heart Age.” This was achieved without the recourse to expensive biomarker tests. However, the results from this study suggest that the motivated lifestyle changes improved both healthy lifestyle risks and psychosocial well-being, consistent with the approach of Life Age in merging the importance of a healthy lifestyle and psychosocial well-being. Further evaluation using a larger randomized controlled trial is required to fully evaluate the impact of the Life Age tool on lifestyle changes, cardiovascular disease prevention, and overall psychosocial well-being.

## Introduction

### Background

Age-based approaches to risk communication have become popular in recent years. The concept of “Heart Age” was previously developed in 2007 [1] to enhance the perception of personal cardiovascular risk in those who would benefit from lifestyle change [2], thereby leading to risk factor reduction [3]. Heart Age has subsequently been adopted by national health organizations [4,5], and its novelty is in being rooted in validated risk models but with an output format that is engaging for users. However, the requirement for biometric factors (eg, blood cholesterol, blood glucose, and blood pressure assessments) in risk models can prevent widespread adoption and potentially lead to misclassification of risk [6].

### Development of the “Life Age” concept

Using lifestyle factors, as opposed to clinical risk factors, in age-based tests could be more useful in promoting cardiovascular health than clinical risk factors in the wider population. In addition to communicating disease risks, communicating on psychosocial well-being factors that constitute the broader lifestyle (life satisfaction, perceived stress, insomnia, and positive and negative mood) may be of equal importance.

Several large observational studies across diverse populations have demonstrated the additive impact of a common set of lifestyle factors on all-cause mortality [7]. Thus, we developed a lifestyle-based assessment of mortality risk by combining published relative risks of all-cause mortality for body weight, physical activity, alcohol, adherence to a Mediterranean diet, and smoking into an overall lifestyle risk score. Relative risks were converted to “effective age” scores for each lifestyle factor using the method devised by Spiegelhalter et al [8] and represented the modifiable mortality risk component of “Life Age.” The range of years that could feasibly be added or removed from a person’s age ranged from –6 years (BMI<23, nonsmoker, no or low alcohol, optimal diet, and high physical activity) to +28 years (BMI>40, smoker, binge drinker, poor diet, and sedentary lifestyle). However, among previous web-based users (n=2000, unpublished), 95% of data fell within a range of –16 to +16 years.

To create the psychosocial well-being component of Life Age, questionnaire scores for life satisfaction, positive and negative mood, hours of sleep, and perceived stress were converted into years (ranging from –2 to +2 years) and based on the distance of score from population norms. This ensured that Life Age scores for psychosocial well-being equalled the impact of healthy lifestyle factors.

### Objective

The aim of this study was to evaluate the motivational potential of a novel lifestyle risk assessment (Life Age) based on factors predictive of both premature mortality and psychosocial well-being.

## Methods

### Study Design

A pilot nonrandomized, single-arm, repeated measures feasibility study (unpublished) on postgraduate students at Imperial College London (London, United Kingdom) examined the impact of taking the web-based version of the Life Age questionnaire without any intervention. We observed a 1.3-year improvement in participants’ Life Age after 8 weeks (*P*=.006). To further evaluate the impact of Life Age on lifestyle change, we conducted a feasibility study using a nonrandomized interventional, single-arm, repeated measures design.

### Ethics Approval

Ethics approval was provided by the Imperial College Research Ethics Committee on April 25, 2018 (18IC4516). The study was carried out in accordance with the tenets of the Declaration of Helsinki. All participants provided consent for data follow-up and publication.

### Participant Recruitment, Newsletters, Mobile Tracker, and Web-Based Feedback Assessment

Participants were recruited via social media. Interested participants completed a web-based version of the Life Age questionnaire at baseline and at follow-up (8 weeks apart). They received 23 e-newsletters based on their Life Age results through email (between weeks 1 and 7) along with a downloadable mobile tracker. The newsletters were developed by the “Younger Lives” expert team, and content was standardized, validated, and centered around the following: (1) setting up participants’ personalized targets on the basis of their initial Life Age score and how to achieve these targets; (2) setting up their environment; that is, setting up the kitchen, particularly a Mediterranean style diet also while eating out, <14 units of weekly alcohol intake, exercising by doing at least 150 minutes of moderate-intensity activity or at least 75 minutes of vigorous-intensity activity weekly [9], self-monitoring using bathroom scales and tape measures for weight and waist measurements, respectively, fitness tracker pedometers on mobile phones or wrist watches, and setting up the bedroom for a good sleep routine; (3) working on daily step counts, diet, being appreciative of good things in life, and understanding and protecting one’s emotional well-being; (4) advice on daily tracking of activities to create lifelong habits; (5) advice on the importance of positive thinking; (6) advice on tips to managing stress at nights; and (7) advice on maximizing health and happiness. Based on their Life Age, the newsletters were formulated to stimulate lifestyle changes based on the distance from recommended lifestyle behaviors in conjunction with their psychosocial well-being; these were provided to encourage them to “get younger” through lifestyle change, and the mobile tracker helped to monitor progress.

The downloadable mobile tracker helped to simplify overall progress tracking. It sets targets on the basis of participants’ Life Age results and maintains a daily log of participants’ body weight, step counts, activity, and sleep. However, participants had to complete a quick 1-minute check-in at the end of each day and also record their weight and waist circumference once a week. The application also helps them to understand if their overall lifestyle has been aging or helping to keep them “young.”

Participants’ estimated Life Age scores were analyzed at baseline and at follow-up for lifestyle change and whether change in health was related to change in psychosocial factors. A quantitative feedback assessment using a web-based questionnaire on ease of use and understanding, motivation, and self-reported lifestyle changes was also conducted.

### Sample Size and Statistical Analysis

The sample size calculation used for the previous pilot study was based on the assumption that pre- and post–2-month intervention might change the mean Life Age by approximately 0.5 years, and the range of change from the participant who decreased his/her Life Age the most and the least is 3 years. Based on these estimates, the sample size was calculated using an SD of 0.75 with a significance level of 5% and power of 90%, and a sample size of 30 participants was agreed on after correcting for a 20% estimated dropout rate. The same sample size was thus used in this feasibility study.

Statistical analysis was performed using Stata software (StataCorp). Parametric and nonparametric data were analyzed using a paired *t* test and Wilcoxon signed-rank test, respectively. A paired *t* test was used to assess differences among Mediterranean diet score, life satisfaction, and combined mood score, whereas the Wilcoxon signed-rank test was used to assess the differences in Life Age, BMI, body weight, physical activity, perceived stress level, and sleep.

The Mediterranean diet score is based on a 14-item score, with a low adherence score being <7, and a higher adherence score being ≥8 according to the Prevención con Dieta Mediterránea (PREDIMED) trial [10]. With regard to mood, higher the positive mood score, better the outcome, and vice versa for negative mood score. The positive mood scores were based on a scale from 1 to 5 points, and its components include being proud of oneself, alert, inspired, determined, attentive, active, interested, excited, strong, and enthusiastic. The negative mood scores were also based on a scale from 1 to 5 points, and its components include being irritable, ashamed, nervous, jittery, afraid, distressed, upset, guilty, scared, and hostile. However, owing to the need for a combined mood variable within our statistical analysis, we proposed the following formula: combined mood = positive mood + (negative mood × –1).

Throughout the data analysis, a *P* value of ≤.05 was considered statistically significant.

### Inclusion Criteria

Participants aged between 30 and 60 years, who signed the consent form, completed the web-based Life Age test, provided self-reported body measurements, have a good understanding of the English language, live within the United Kingdom, and use an iPhone owing to mobile tracker compatibility were included in the study.

## Results

### Sample Characteristics

Between April and May 2018, a total of 27 eligible individuals were enrolled in the study at baseline. At baseline, their average chronological age was 37 years, Life Age was 1 year older than the chronological age, BMI was 24.2 kg/m², waist circumference was 81.3 cm, body weight was 68 kg, amount of physical activity per week was 13.3 metabolic equivalents of task (METS) per hour, and 17 (63%) of them were female. Other baseline characteristics are shown in Table 1.

**Table 1:** Characteristics of participants between baseline and follow-up.

Characteristics			Baseline (n=27)		Follow up (n=18)		*P* value
Female sex, n (%)			17 (63)		13 (72)		N/A^a^
Age (years), median (IQR)			37 (30 to 56)		37 (30 to 57)		N/A
BMI (kg/m²), median (IQR)			24.2 (19.4 to 34.7)		22.8 (19.5 to 31.8)		.21
BMI<25 kg/m^2^, n (%)			15 (56)		12 (67)		N/A
BMI=25-29.9 kg/m^2^, n (%)			7 (26)		4 (22)		N/A
BMI>30 kg/m^2^, n (%)			5 (18)		2 (11)		N/A
Waist circumference (cm), median (IQR)			81.3 (67 to 111)		77.5 (50 to 101.6)		.20
Weight (kg), median (IQR)			68 (51 to 116)		65 (50 to 93)		.18
**Smoking status, n (%)**							
	Never smoked	20 (74)		15 (83)		N/A	
	Ex-smoker	7 (26)		3 (17)		N/A	
	Current smoker	0 (0)		0 (0)		N/A	
Nutrition (units), mean (SD)			7.1 (1.8)		8.5 (1.8)		.001
Weekly physical activity, median (IQR)			13.3 (0 to 43.3)		16.7 (0 to 38)		.12
Life age (years), median (IQR)			1 (–5 to 12.75)		–1.9 (–6.3 to 11.5)		.02
Life satisfaction (units), mean (SD)			30.6 (6.3)		34.2 (5.2)		.003
Stress level, median (IQR)			15 (5 to 27)		14 (9 to 31)		.06
Sleep (hours), median (IQR)			6.5 (4.5 to 8)		7 (5 to 8)		.05
**Mood score**							
	Positive mood, mean (SD)	35.7 (6.4)		37.5 (6.0)		.22	
	Negative mood, median (IQR)	17 (10 to 36)		15.5 (10 to 27)		.21	

### Main Findings

In total, 18 of 27 participants completed both Life Age tests, which were separated by an 8-week interval. At follow-up, the median Life Age was –1.9 years, which was younger than the chronological age, representing a 2.9-year reduction in Life Age over 8 weeks (*P*=.02). An analysis of individual risk factor components revealed an improvement of 1.4 units in the Mediterranean diet score (*P*=.001), 3.6-unit increase in life satisfaction (*P*=.003), and a 0.5-hour increase in sleep (*P*=.05), whereas perceived stress levels improved to a degree approaching statistical significance (*P*=.06; Table 1). Clinically relevant improvements were observed in BMI (1.4 kg/m²), waist circumference (3.8 cm), body weight (3 kg), physical activity (3.4 METS per hour), and mood (3.3 units); however, these failed to reach the preassigned level of statistical significance. There was a high dropout rate, which resulted in a smaller sample at the end of the study, which affected some secondary outcomes including BMI, weight, physical activity, and mood.

### Web-Based Feedback Assessment Findings

Web-based feedback assessment from 16 of 18 participants who completed the study revealed that 10 of 16 (63%) participants felt that the lifestyle recommendations provided were helpful, 9 (56%) would recommend the Life Age tool to their friends and family, 11 (69%) found the mobile tracker easy to understand and user friendly, 9 (56%) found the newsletters informative and user friendly, 11 (69%) would recommend the use of the mobile tracker and newsletter to their friends and family, 11 (69%) were motivated to change their lifestyle via the use of newsletters, 12 (75%) found the dietary advice within the newsletters most useful, and 11 (69%) felt confident to continue with their lifestyle changes after the study ended.

## Discussion

### Principal Findings and Comparison With Previous Work

This feasibility study explored the development and impact of a novel lifestyle risk assessment tool, Life Age, on lifestyle changes. We observed a significant improvement in the overall Life Age metric, adherence to Mediterranean diet, life satisfaction, and sleep. Furthermore, there were clinically relevant improvements in perceived stress level, BMI, waist circumference, body weight, physical activity, and mood, but these failed to reach the preassigned level of statistical significance, in part owing to high variability at baseline and lack of power in the study. The improvement in Life Age was in agreement with the findings of the initial pilot study in 2017 (unpublished), which reported a median improvement of 1.3 years (*P*=.006).

### Comparison Between Different Age-Based Approaches: Life Age and Heart Age

Although Heart Age was developed to enhance cardiovascular risk perception with the aim of facilitating lifestyle changes and subsequent risk factor reduction, it has been adopted by national health organizations [1-5]; however, a recently published systematic review of 16 studies assessed the effects of Heart Age intervention and reported that absolute risk could be reduced but with minimal evidence that it motivates lifestyle behavior. When combined with behavioral change strategies, there is evidence that it can improve clinical outcomes [11]. In comparison, this feasibility study on Life Age reported significant lifestyle behavior changes, particularly in the adherence to a Mediterranean diet and improvement in some psychosocial factors such as life satisfaction and sleep facilitated by the use of e-newsletters and a mobile tracker.

### Comparison With Known Evidence on Mediterranean Diet, Life Satisfaction, and Sleep

Adherence to a Mediterranean diet was encouraged throughout this study, and there was an improvement in the Mediterranean diet score by 1.4 units from 7.1 to 8.5 units (*P*=.001). A score of ≥8 indicates a higher level of adherence to the Mediterranean diet [10]. Evidence from the PREDIMED trial and the Health, Alcohol and Psychosocial factors In Eastern Europe (HAPIEE) study have demonstrated that increased adherence to this diet reduces major adverse cardiovascular events [12,13]. Similarly, there was an improvement in life satisfaction score by 3.6 units from 30.6 to 34.2 units (*P*=.003) at the end of this study. Few studies have shown an association between low life satisfaction and an increased risk of cardiovascular disease [14,15]. Furthermore, there was an improvement in average sleep duration from 6.5 hours to 7 hours at the end of this study (*P*=.05). Evidence from systematic reviews has shown that both short and long sleep durations (<7 hours and >7 hours, respectively) are associated with an increased risk of cardiovascular events and all-cause mortality [16,17].

### Limitations and Strengths

A limitation in this study was the potential for under- or overestimation of self-reported body measurements. Attempts to minimize the impact of measurement bias was addressed by asking participants to provide measurements of recent visits to general practitioners, surgeons, or the gymnasium. A further limitation was a higher dropout rate and a smaller sample size at the end of the trial, which affected some secondary outcomes such as BMI, weight, physical activity, and mood. However, as this was a feasibility study, these limitations are likely to be addressed in a larger randomized trial. Finally, the total sample is small, and participants reported health parameters that were healthier than the average population, potentially limiting the generalizability of this study. Nevertheless, this study has several strengths: first, minimal resources were used in this feasibility study, thus making it cost-effective at this stage of development. Second, the use of lifestyle assessments instead of clinical risk factors reduced the burden on participants in this feasibility study and no harm was encountered. Third, although this was the second study evaluating the impact of the use of this novel Life Age tool, it is the first to combine the Life Age assessment with follow-up material such as newsletters and mobile tracker. This enabled us to evaluate the longer-term impact on participant behavior. Finally, feedback assessment showed that a significant proportion of the participants found the intervention to be useful and user friendly.

### Future Direction

Based on the findings of this feasibility study, we propose a larger randomized controlled trial to fully evaluate the longer-term impact of the Life Age tool on lifestyle changes and risk factors, in addition to a head-to-head comparison with risk factor–based tests such as Heart Age to understand whether lifestyle factors or clinical risk factors are modified equally or differently by different approaches.

### Conclusions

This study demonstrated the potential benefit of a novel Life Age tool in generating a broad set of lifestyle changes known to be associated with clinical risk factors similar to Heart Age. This was achieved without the recourse to expensive biomarker tests. However, results from this study suggested that the motivated lifestyle changes improved both healthy lifestyle risks and psychosocial well-being, consistent with the approach of Life Age in merging the importance of a healthy lifestyle and psychosocial well-being. Further evaluation using a larger randomized controlled trial is required to fully evaluate the impact and relative merit of the Life Age tool on lifestyle changes, cardiovascular risk factors, and overall psychosocial well-being. Comparison of this assessment versus commonly used risk assessments that include biomarkers can help identify the value associated with the noninvasive approach to risk assessment.

## Abbreviations

HAPIEE: Health, Alcohol and Psychosocial factors In Eastern Europe
METS: metabolic equivalents of task
PREDIMED: Prevención con Dieta Mediterránea

## Data Availability

The data sets generated or analyzed during this study are available on request from the corresponding author, these data are not publicly available owing to privacy and ethical restrictions.
